# Nephroureterectomy of Right-to-Left Crossed Fused Renal Ectopia with Urothelial Carcinoma

**DOI:** 10.7759/cureus.8544

**Published:** 2020-06-10

**Authors:** Jason Hearn, Rebecca J Power, Landan MacDonald, Paul Johnston, Michael Organ

**Affiliations:** 1 Urology, Memorial University of Newfoundland, St. John's, CAN; 2 Urology, Dalhousie University, Halifax, CAN

**Keywords:** urology, urologic oncology, crossed fused renal ectopia, congenital anomaly, urothelial carcinoma, transitional cell carcinoma, nephroureterectomy

## Abstract

Urothelial carcinoma in a crossed fused renal ectopia (CFRE) is an exceedingly rare clinical finding. We describe the surgical management used to treat upper tract urothelial carcinoma in a 64-year-old man with a right-to-left CFRE. Nephroureterectomy with bladder cuff excision was the treatment of choice. The fused kidney was carefully dissected until an area of demarcation emerged between the vasculature supplying the left and right moieties. Pressure was applied to the isthmus separating the two moieties, and a sharp incision was made to release the left moiety. The operation was completed with limited blood loss. Pathology revealed a high-grade T3 papillary urothelial carcinoma with negative margins. To our knowledge, the case is the first to report urothelial carcinoma in a right-to-left CFRE.

## Introduction

Crossed fused renal ectopia (CFRE) is a rare anatomic anomaly in which an individual’s kidneys are fused together and lie on the same side of their midline [1]. Upper tract urothelial (or transitional cell) carcinomas arise in the renal pelvis or ureter and account for less than 10% of all renal carcinomas [2]. The coincidence of these two pathologies is exceedingly rare, with only two prior cases identified in the literature [3-4]. Thus, the optimal surgical technique indicated in cases of CFRE with urothelial carcinoma remains poorly understood. We present the first known case in which a nephroureterectomy was used to treat an upper tract urothelial carcinoma in an individual with a right-to-left CFRE.

## Case presentation

**Figure 1:**
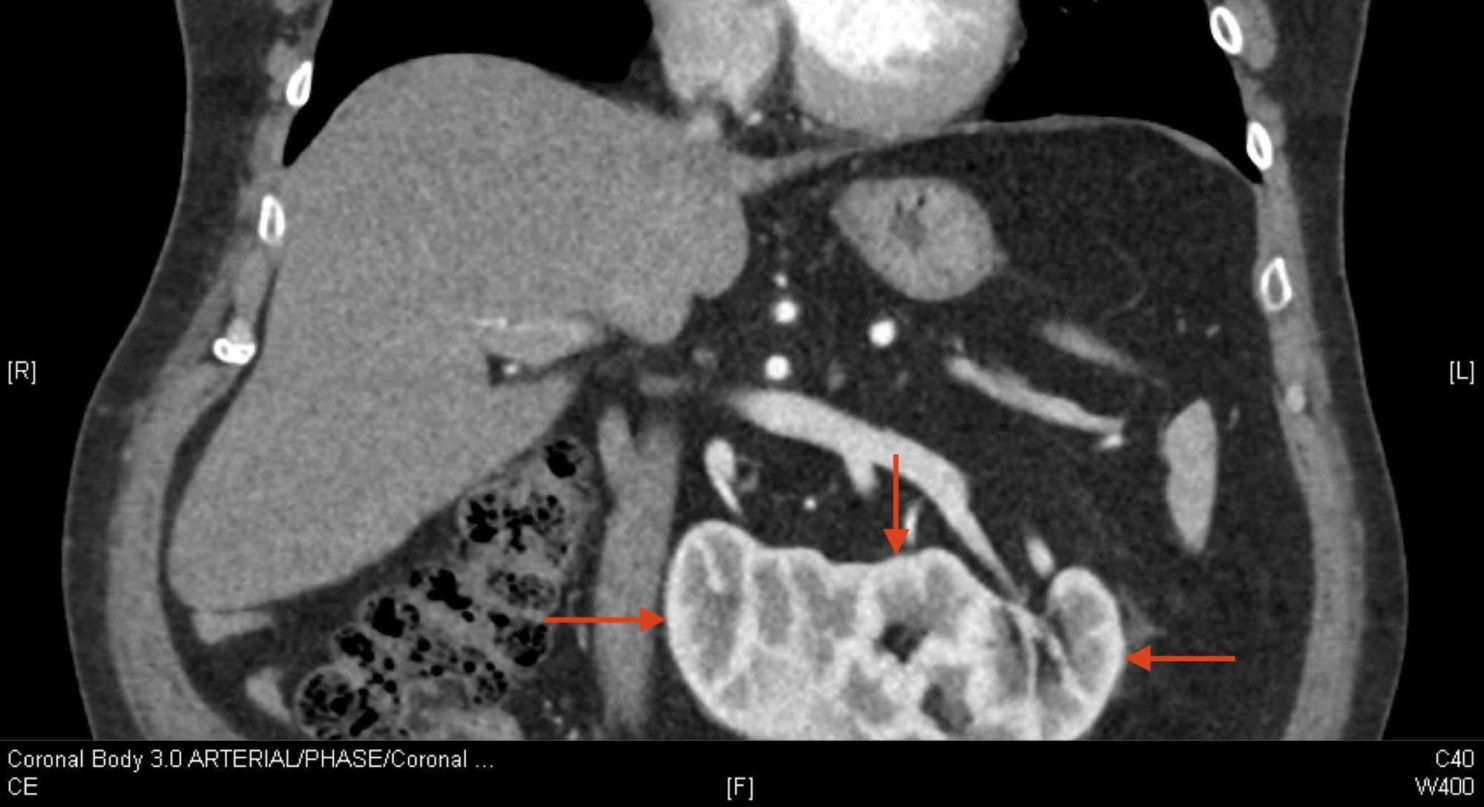
Computerized tomography urogram demonstrating the crossed fused renal ectopia

**Figure 2:**
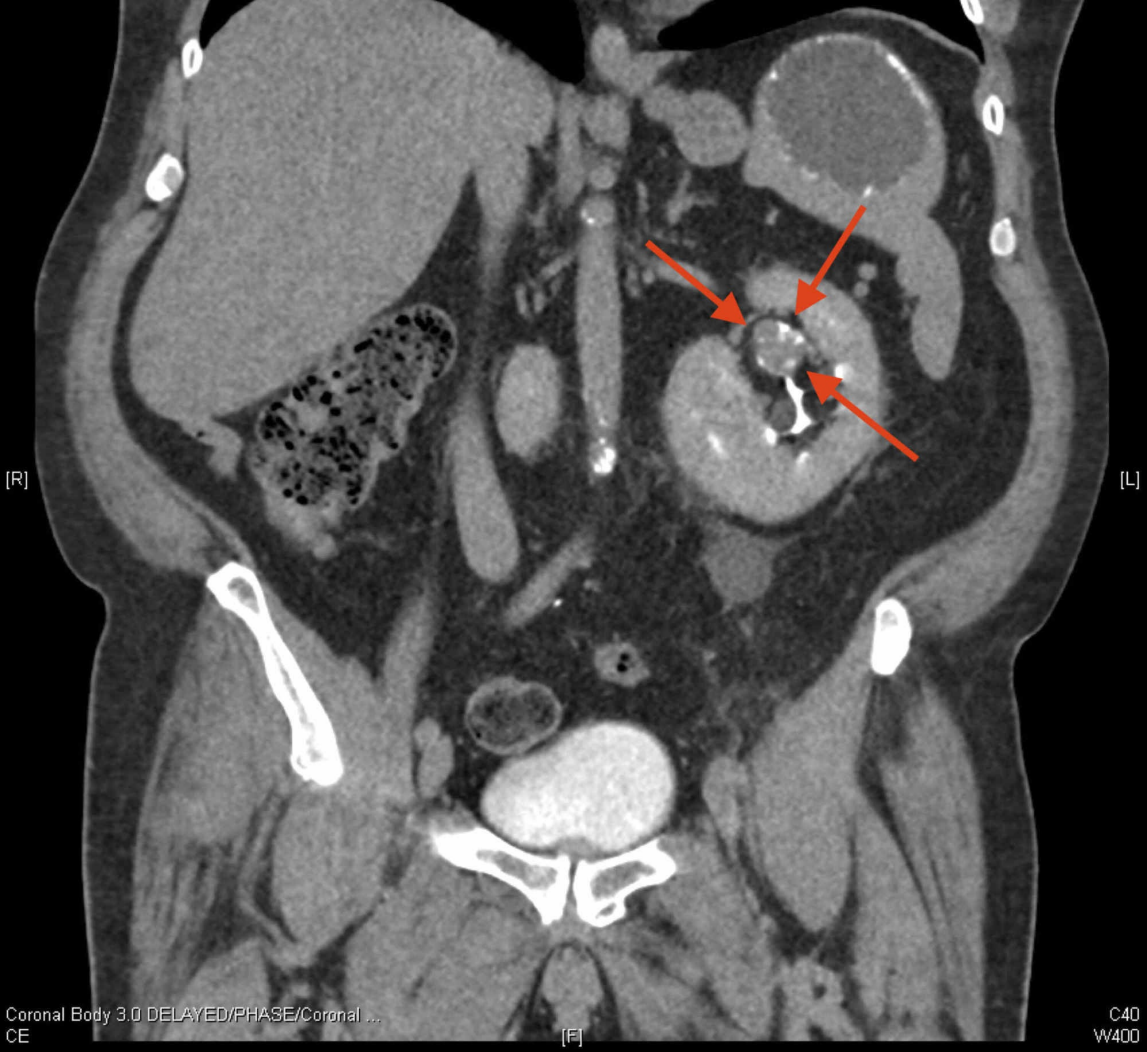
Computerized tomography urogram demonstrating a filling defect in the left upper pole of the fused kidney

A 64-year-old male presented with gross hematuria. His past medical history was significant for coronary artery disease, obstructive sleep apnea, type 2 diabetes, hypertension, erectile dysfunction, and surgery for diverticulosis. Recent investigations revealed a creatinine level of 104 µmol/L and a glomerular filtration rate greater than 60 mL/min/1.73 m2. A computerized tomography urogram revealed a right-to-left CFRE, which roughly approximated a horseshoe kidney (Figure 1). The urogram also identified a filling defect in the left upper pole of the fused kidney (Figure 2). The renal tissue was biopsied using videoscopic ureteroscopy and pathology identified a high-grade pTa lesion. Given the stage and location of the lesion, the chosen treatment was nephrectomy of the left moiety with ureterectomy and bladder cuff excision. 

The patient was positioned in the supine position with exposure of both the lower abdomen and the penis. A flexible cystoscope was introduced via the urethra and ureteral stents were placed inside both the left and right moieties of the kidney, allowing for accurate differentiation of the two collecting systems. Next, a large midline incision was made from the umbilicus to the pubic symphysis. The descending colon and small intestine were then mobilized and reflected to expose the fused kidney and the left renal vein.

Two branches from the left renal vein to the kidney were identified: one extending to the left moiety and the other to the right moiety. The left renal artery was then identified posterior to the renal vein. Accessory arterial branches were dissected, and ligation was placed just distal to the single branch supplying the right moiety of the kidney. The veins corresponding to the left moiety were then ligated and dissected. Finally, the portion of the artery distal to the ligation was dissected, providing full control of the left moiety vasculature. Next, the kidney was skeletonized until a clear area of demarcation emerged between the vasculature of the two moieties. Pressure was applied to the isthmus separating the two moieties to achieve reasonable hemostasis, and a sharp incision was made to release the left moiety from the right. Blood loss at this stage was minimal, as both gross and parenchymal bleeding were quickly controlled.

Ureterectomy and bladder cuff excision were subsequently performed without any difficulties. Upon reassessment of the kidney, there was no evidence of bleeding in the hilum and the remaining moiety appeared to be well-perfused. A node dissection was briefly considered but was ultimately not performed given the complexity of the vasculature and the risk to the remaining portion of the kidney. Overall, the surgery was completed without complications and the estimated blood loss was 1400 mL.

The final pathology revealed a high-grade T3 papillary urothelial carcinoma with negative margins. On gross examination, the tumor appeared to invade through the renal calyces into the parenchyma. The lesion was located more than 2.5 cm from the renal vein and did not appear to extend into the renal sinus fat. Microscopic evaluation revealed no evidence of lymphovascular invasion.

Postoperatively, the patient recovered well despite the size and complexity of the surgery. In the weeks following the operation, he developed left-sided testicular pain and swelling consistent with epididymitis. The presumed infection was treated with ciprofloxacin. A scrotal ultrasound was also performed to confirm the benignity of the testicular symptoms. Postoperative investigations revealed a glomerular filtration rate of 46 mL/min/1.73 m2 (at 40 days) and a creatinine clearance of 67 mL/min (at 66 days). Adjuvant gemcitabine and cisplatin chemotherapy was initiated at three months following the operation and continued for four 21-day cycles. At nine months, the patient was doing well with no evidence of metastatic disease on clinical examination or imaging. Investigations at this time revealed a creatinine level of 169 µmol/L and a creatinine clearance of 57 mL/min.

## Discussion

CFRE is a congenital malformation with an estimated incidence of one in 1000 live births [5]. It is hypothesized that CFRE is associated with improper development of the ureteric bud and/or the nephrogenic blastema, though the exact causal mechanism remains unknown [6]. The abnormality has a 3:2 predominance in males, and left-to-right crossover of the kidneys is three times more common than right-to-left crossover [7]. Evidently, CFRE presents several challenges when performing a nephroureterectomy, as the abnormal anatomy necessitates careful consideration of the renal vasculature and accurate dissection of the renal parenchyma [3].

Urothelial carcinoma of the kidney is also relatively uncommon, as upper urinary tract lesions account for only 5%-7% of all urothelial carcinomas [8]. The defining features of urothelial carcinomas include multiplicity and a high incidence of recurrence [9]. Due to these characteristics, the recommended treatment for urothelial carcinoma of the kidney is nephroureterectomy with excision of a bladder cuff. Moreover, given the likelihood of invasion into the bladder, regular cystoscopic follow-up is encouraged [10].

To our knowledge, only two other cases of upper tract urothelial carcinoma in an individual with CFRE have been reported in the literature. The first reported case involved a patient with left-to-right CFRE and invasive urothelial carcinoma of the kidney. The operating surgeons performed an open nephroureterectomy with bladder cuff resection, and the subsequent pathology identified a high-grade T1 carcinoma of the ureter [3]. The second case also involved upper tract urothelial carcinoma in an individual with left-to-right CFRE. However, surgical intervention was performed laparoscopically and the pathology revealed a low-grade T1 carcinoma [4]. Based on this review of the literature, we present the first reported case of urothelial carcinoma in a right-to-left CFRE, as well as the most advanced case of upper tract urothelial carcinoma in a patient with CFRE. Moreover, we present only the second instance in which an open nephroureterectomy was used to treat upper tract urothelial carcinoma in a patient with CFRE.

## Conclusions

The co-occurrence of both CFRE and upper tract urothelial carcinoma is a clinical rarity. We report the case of a 64-year-old male presenting with high-grade urothelial carcinoma in the upper left pole of a right-to-left CFRE. Based on initial assessments, the chosen treatment was nephrectomy of the left moiety with ureterectomy and bladder cuff excision. With careful consideration of the renal vasculature, the operation was completed without complications and with limited blood loss. The case is the first to report urothelial carcinoma in a right-to-left CFRE and only the second to describe open nephroureterectomy of a CFRE with urothelial carcinoma.
